# Effects of tetrahedral DNA nanostructures on the treatment of osteoporosis

**DOI:** 10.1111/cpr.13625

**Published:** 2024-02-27

**Authors:** Weitong Cui, Xiao Yang, Yikai Dou, Yue Du, Xiaohong Ma, Lei Hu, Yunfeng Lin

**Affiliations:** ^1^ State Key Laboratory of Oral Diseases, National Center for Stomatology, National Clinical Research Center for Oral Diseases, West China Hospital of Stomatology Sichuan University Chengdu China; ^2^ Psychiatric Laboratory and Mental Health Center, the State Key Laboratory of Biotherapy West China Hospital of Sichuan University Chengdu China; ^3^ Department of Orthopedics Sichuan Langsheng Brain Hospital & Shanghai Langsheng Brain Hospital Investment Co., Ltd. Chengdu China; ^4^ Sichuan Provincial Engineering Research Center of Oral Biomaterials Chengdu China; ^5^ National Center for Translational Medicine Shanghai Jiao Tong University Shanghai China

## Abstract

Osteoporosis (OP) is a common disease characterized by bone loss and bone tissue microstructure degradation. Drug treatment is a common clinical treatment that aims to increase bone mass and bone density. Tetrahedral DNA nanostructures (TDNs) are three‐dimensional tetrahedral frames formed by folding four single‐stranded DNA molecules, which have good biological safety and can promote bone regeneration. In this study, a mouse model of OP was established by ovariectomy (OVX) and TDN was injected into the tail vein for 8 weeks. We found that ovariectomized mice could simulate some physiological changes in OP. After treatment with TDNs, some of this destruction in mice was significantly improved, including an increase in the bone volume fraction (BV/TV) and bone trabecular number (Tb. N), decrease in bone separation (Tb. SP), reduction in the damage to the mouse cartilage layer, reduction in osteoclast lacunae in bone trabecula, and reduction in the damage to the bone dense part. We also found that the expression of ALP, β‐Catenin, Runx2, Osterix, and bone morphogenetic protein (BMP)2 significantly decreased in OVX mice but increased after TDN treatment. Therefore, this study suggests that TDNs may regulate the Wnt/β‐Catenin and BMP signalling pathways to improve the levels of some specific markers of osteogenic differentiation, such as Runx2, ALP, and Osterix, to promote osteogenesis, thus showing a therapeutic effect on OP mice.

## Graphical abstract

Tetrahedral DNA nanostructures regulate the Wnt/β⁃Catenin and bone morphogenetic protein signalling pathways to promote osteogenesis, thus showing a therapeutic effect on osteoporosis mice.

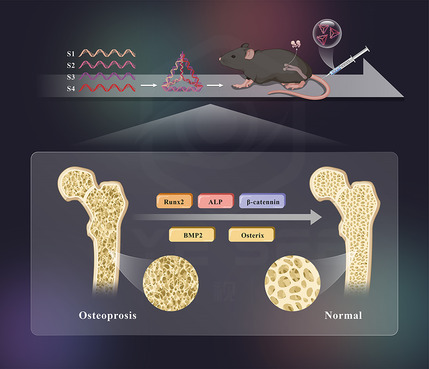

## 1 BACKGROUND

Osteoporosis (OP) is a common and serious bone‐related disease.^1^ This disease is prone to occur in postmenopausal women, is prone to cause many complications, affects individual daily life, and may even increase the risk of individual death.^2^, ^3^ The main pathological features of the disease are bone loss and bone tissue microstructure degeneration.^4^ Individual bone metabolism is a dynamic balance process of bone reconstruction, in which osteoblasts synthesize bone matrix to lead bone formation, and osteoclasts absorb bone matrix to lead bone absorption. The dynamic balance between bone formation and bone absorption is essential to maintain the function and shape of normal bone tissue. If this balance is disrupted, it will lead to OP, delayed fracture healing, and other bone‐related diseases.^5^

Drug treatment is a common treatment for OP, with the purpose of increasing individual bone mass and bone density.^6^ However, the long‐term use of such drugs has uncertain long‐term efficacy and side effects, such as an increased risk of cardiovascular events.^7^, ^8^, ^9^ Therefore, researchers gradually began to explore other drugs that can improve OP with few side effects. In the population with OP, the reduction in bone density and the reduction in fracture healing ability may be related to the reduction in bone marrow mesenchymal stem cell (MSC) proliferation and osteogenic differentiation ability.^10^ Therefore, researchers have been committed to finding drugs that can promote their differentiation ability and reduce bone loss and fat formation in the OP bone marrow cavity.

In recent years, with the development of nanomaterials, researchers have found that some nucleic acid nanomaterials also have certain effects on bone regeneration.^11^, ^12^ For example, tetrahedral DNA nanostructures (TDNs) are three‐dimensional tetrahedral frame structures formed by folding four single‐stranded DNA molecules.^13^ Previous studies have found that TDNs can regulate the biological behaviour of cells, such as promoting cell proliferation and regulating stem cell differentiation, including adipose stem cells, neural stem cells, dental pulp stem cells, and other stem cells,^11^, ^14^ all of which suggest that TDNs are a good stem cell synergist.^12^, ^15^ Our previous research showed that TDNs can regulate the Wnt/β‐Catenin pathway and promote the osteogenic differentiation of MSCs, thus playing a role in the treatment of osteoarthritis,^16^ which has also been confirmed in an in vitro study.^17^ However, the underlying mechanism of the effects of TDNs in promoting bone repair in OP is still unclear.

Therefore, in this study, we established a mouse model of OP and used TDNs to treat it. To observe the effect of TDNs on OP and explore its potential mechanism. Based on our previous studies, we speculate that TDNs may activate the Wnt/β‐Catenin pathway or bone‐induced growth factors, such as bone morphogenetic protein (BMP), and can promote the osteogenic differentiation of MSCs in an OP animal model, thus playing a role in the treatment of OP.

## 2 MATERIALS AND METHODS

### 2.1 Synthesis and characterization of TDNs

The sequences of the four single strands used are given in Table 1. According to the previous scheme of our group, four single strands were dissolved in Tm buffer (10 mmol/L Tris HCl, 50 mmol/L MgCl_2_ · 6H_2_O, pH 8.0), heated to 95°C for 10 min, and cooled to 4°C for 20 min. Polyacrylamide gel electrophoresis (PAGE) was used to verify the successful synthesis of TDNs. Transmission electron microscopy (TEM) and atomic force microscopy (AFM) were used to detect the spatial structure and shape of the TDNs. Then, dynamic light scattering (DLS) was used to detect the particle size and potential of the TDNs.

**TABLE 1:** Base sequences of each single‐stranded DNA (ssDNA).

ssDNA	Base sequence (5′ → 3′)
S1	ATTTATCACCCGCCATAGTAGACGTATCACCAGGCAGT
	GAGACGAACATTCCTAAGTCTGAA
S2	ACATGCGAGGGTCCAATACCGACGATTACAGCTTGCT
	ACACGATTCAGACTTAGGAATGTTCG
S3	ACTACTATGGCGGGTGATAAAACGTGTAGCAAGCTGT
	AATCGACGGGAAGAGCATGCCCATCC
S4	ACGGTATTGGACCCTCGCATGACTCAACTGCCTGGTG
	ATACGAGGATGGGCATGCTCTTCCCG

### 2.2 Animal models

Ovariectomy (OVX) is a classic animal model for studies of OP that can successfully simulate the characteristics of bone metabolism in postmenopausal OP.^18^ In this experiment, we randomly divided 30 8‐week‐old female ICR mice (Changzhou Cavens) into five groups: Group 1, sham operation group (Sham); Group 2, ovariectomized model group (OVX group); and Groups 3–5, ovariectomized model group (OVX group) with different doses of drug treatment (250, 500, and 1000 nM TDNs, respectively). The number of mice in each group was equal. The ovariectomized mice were anaesthetized with 1% pentobarbital sodium solution and injected intraperitoneally at a concentration of 75 mg/kg. A small incision of ~1 cm was made at the waist of the midline of the abdomen of the mouse, the subcutaneous tissue was passively separated, the ovary was carefully removed, and the blood vessels and tissues on both sides of the ovary were electrocoagulated. The ovaries were removed and sutured layer by layer, and 100 μL penicillin (3000–3500 U) was added. In Group 1, after the ovaries were separated under anaesthesia, the skin and flesh were sutured layer by layer as in the sham operation group, and the ovaries were removed in Groups 2–5. After surgical treatment, all animals were disinfected with iodine and put back into cages for breeding.

Then, all animals were injected intraperitoneally once a day for 8 weeks, with an injection volume of 200 μL, in which Groups 1 and 2 were injected intraperitoneally with nucleic acid nanomaterial solvent (TM buffer), and TDNs of 250, 500, and 1000 nM were injected intraperitoneally in Groups 3–5. After 8 weeks of modelling and treatment, the mice were scarified. Then, the mouse eyeballs were removed, and ~1 mL of blood was collected into a 2 mL EP tube and stored at 4 degrees overnight. Then, ~200 μL of supernatant was absorbed and stored at −80°C for subsequent enzyme‐linked immunosorbent assay (ELISA) detection. The experiment on experimental animals was approved by the Ethics Committee of Sichuan University and conformed to the relevant laws on experimental animals.

### 2.3 Microcomputed tomography

The left femurs of all animals were collected, and the surrounding muscle tissue of the left femurs of mice was gently removed. Each group had six samples, and three samples were soaked in 4% paraformaldehyde for microcomputed tomography (Micro‐CT) examination at 2 months. The remaining three samples were frozen in liquid nitrogen and stored at −80°C for western blot analysis. Each sample was scanned using the Skyscan 1176 Micro‐CT Scanner software (voltage, 70 kV).

### 2.4 Slicing

The tubes with fixed tissue were filled with EDTA decalcification solution, sealed, and placed into a constant temperature shaker. The temperature was between 25 and 30°C, and the shaking rate was between 110 and 120 RPM. The degree of decalcification was observed every 2 days, and the replacement period of decalcification solution was 2–3 days. Then, alcohol was used for gradient dehydration in the dehydrator, and the sections were wrapped in melted paraffin. After that, the wax‐soaked tissue was embedded in the embedding machine and cooled on a −20°C freezing table, and the wax block was removed from the embedding frame and trimmed after the wax solidified. Finally, the sections were sliced in a paraffin sectioning machine at a thickness of 4 μm. The slices were floated in the 40°C warm water of the spreader to flatten the tissue, and then the slides were picked up and baked in a 60°C oven. After drying with water, the wax was removed and stored at room temperature.

### 2.5 Haematoxylin–eosin staining

First, wash the slices with distilled water, then add haematoxylin to dye it, and control the dyeing time according to the tissue and dye conditions, usually ~5 min. Then, the tissue was washed with distilled water until it was blue and purple. Then, 1% hydrochloric acid and ethanol were added to differentiate for 2 s, and the tissue was allowed to turn red. Then, wash the tissue with distilled water again until it is blue and purple. Then, eosin staining was conducted, and the staining time was controlled (usually ~2 s) according to the tissue staining situation. Then, wash the slices with distilled water. After dehydration with anhydrous ethanol, neutral gum was added, and the film was sealed. The slides were observed and photographed under an inverted microscope (OLYMPUS IX71).

### 2.6 Tartrate‐resistant acid phosphatase staining

Tartrate‐resistant acid phosphatase (TRAP) staining was carried out using a TRAP staining kit (Sorebol G1492). Slices were dewaxed in water. Then, the slices were fixed with TRAP fixation solution at 4°C for 1 min and flushed with running water for 1 min. TRAP staining solution at 37°C was added for 60 min. The slices were rinsed again with running water for 1 min. Haematoxylin staining was performed at room temperature for 5 min. Sections were rinsed with running water until the tissue turned blue. The tissue was soaked in anhydrous ethanol, and the liquid around the tissue was dried. Then, the sections were sealed with neutral gum. The positive expression of TRAP staining under a microscope is purplish blue, and the nucleus is blue.

### 2.7 Immunohistochemistry

First, rehydrate the slices and wash them with PBS, repeated three times for 5 min each time. After that, antigen repair was carried out, and the sections were immersed in antigen repair solution in a microwave oven for 15 min. Then, the slices were washed with PBS three times for 5 min each time. Then, slices were incubated with H_2_O_2_ at room temperature for 10 min, rinsed with PBS for 5 min, and repeated three times. Then, slices were incubated with 0.1% Trion X‐100 at room temperature for 10 min, rinsed with PBS for 5 min, and repeated three times. Then, one drop (50 μL) of sealing liquid (5% BSA) was added above the tissue for 1 h at 37°C. Then, the primary antibodies against β‐Catenin (1:100, GB12015), Runx2 (1:300, GB11264), Osterix (1:200, GB111900), ALP (1:100, Gb11527), and BMP2 (1:200, GB11252) were diluted in proportion and added to the tissue separately for 2 h at 37°C. Then, the cells were washed with PBS 3 times for 5 min each time. Then, the sections were incubated with the secondary antibody, diluted in proportion, and added to the tissue for 1 h at 37°C, washed with PBS and repeated three times for 5 min each time. After that, Hochester was dripped onto the tissue at room temperature for 15 min, rinsed with PBS for 5 min and repeated three times. Finally, the water around the tissue was dried, soaked in anhydrous ethanol for 1 min and removed. After the slice was dried, the tissue part on the non‐tissue side of the slide was marked with a marker. Then, the slide was sealed with an anti‐quenching sealing agent and observed and photographed under a fluorescence microscope.

### 2.8 Western blotting

The tissue was ground thoroughly, RIPA lysis buffer and 1/100 PMSF were added to each group, and the samples were placed on ice for 2 h. Then, the samples were centrifuged for 10 min at 12000 rpm and 4°C. The supernatant was transferred into a new EP tube and stored in a refrigerator at −20°C after protein quantification. Protein samples were separated by SDS–PAGE and transferred to PVDF membranes. The membrane was then placed in a closed solution and mixed with the corresponding primary antibodies against β‐Catenin (1:500, Affinity), Runx2 (1:200, Proteintech), Osterix (1:1000, Abcam), ALP (1:1000, Abcam), BMP2 (1:2000, Proteintech), and GAPDH (1:1000, Proteintech) and incubated overnight. The next day, the membrane was washed with TBST five times for 10 min each time. Then, the secondary antibody corresponding to the primary antibody was diluted 1:5000 and incubated with the membrane at 37°C for 1 h. Then, the membrane was washed with TBST five times for 10 min each time. Finally, membranes were incubated with exposure liquid, the ECL illuminator was exposed, and images were collected. The data were processed using ImageJ software.

### 2.9 Enzyme‐linked immunosorbent assay

An ALP ELISA kit (Cloud‐Clone Corp) and calcium (Ca) test kit (built‐in Nanjing) were used to quantify the expression of serum ALP and calcium ions in the supernatants. The OD value was measured at a wavelength of 450 nm, and the concentrations of ALP and calcium ions (Ca^2+^) in the sample were calculated separately through the standard curve. Tests were carried out in triplicate.

### 2.10 Statistical analysis

SPSS 24.0 (IBM, Silicon Valley, CA, USA) was used for the statistical analysis. Student's *t*‐test and one‐way analysis of variance were applied. Quantitative results are presented as the mean ± standard deviation. A *p‐*value <0.05 indicated statistical significance.

## 3 RESULTS AND DISCUSSION

### 3.1 Preparation and characterization of TDNs

**FIGURE 1:**
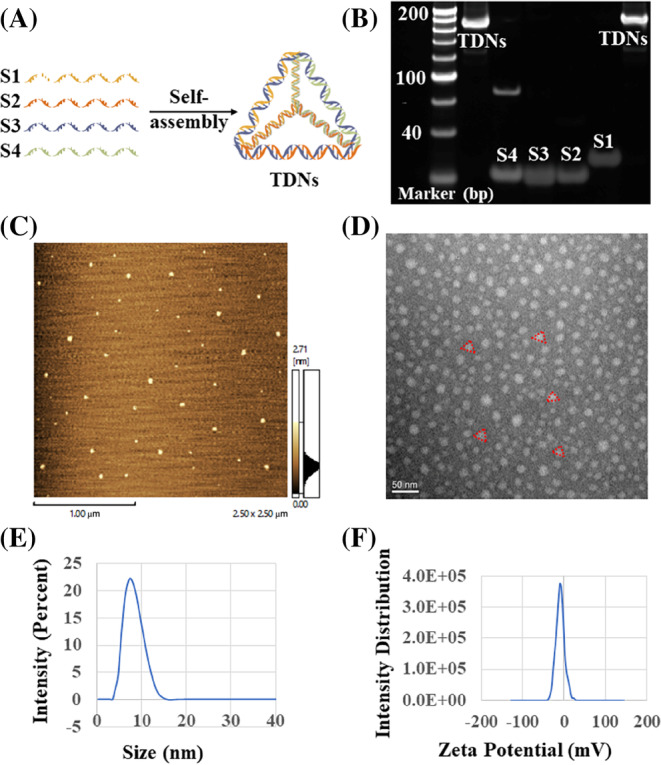
Successful synthesis and characterization of tetrahedral DNA nanostructure (TDN). (A) Schematic diagram of TDNs synthesis. (B) Polyacrylamide gel electrophoresis gel shows the successful synthesis of TDN, Lane 1–6: TDNs, S4, S3, S2, S1 TDNs. (C) Atomic force photo of TDN (Scale bar: 100 nm). (D) Transmission electron microscope photo of TDN (Scale bar: 50 nm). (E,F) Particle size and potential of TDN.

According to the previous methods, four single strands of DNA (S1–S4) were self‐assembled to synthesize TDNs (Figure 1A), and the successful synthesis of TDNs was verified by PAGE (Figure 1B). TEM and AFM detected the spatial structure and shape of the TDNs, respectively (Figure 1C,D). Through DLS measurements, the size of the TDNs is ~7.53 nm. The zeta potential is −6.28 mV (Figure 1E,F).

### 3.2 OVX‐induced OP mouse model

**FIGURE 2:**
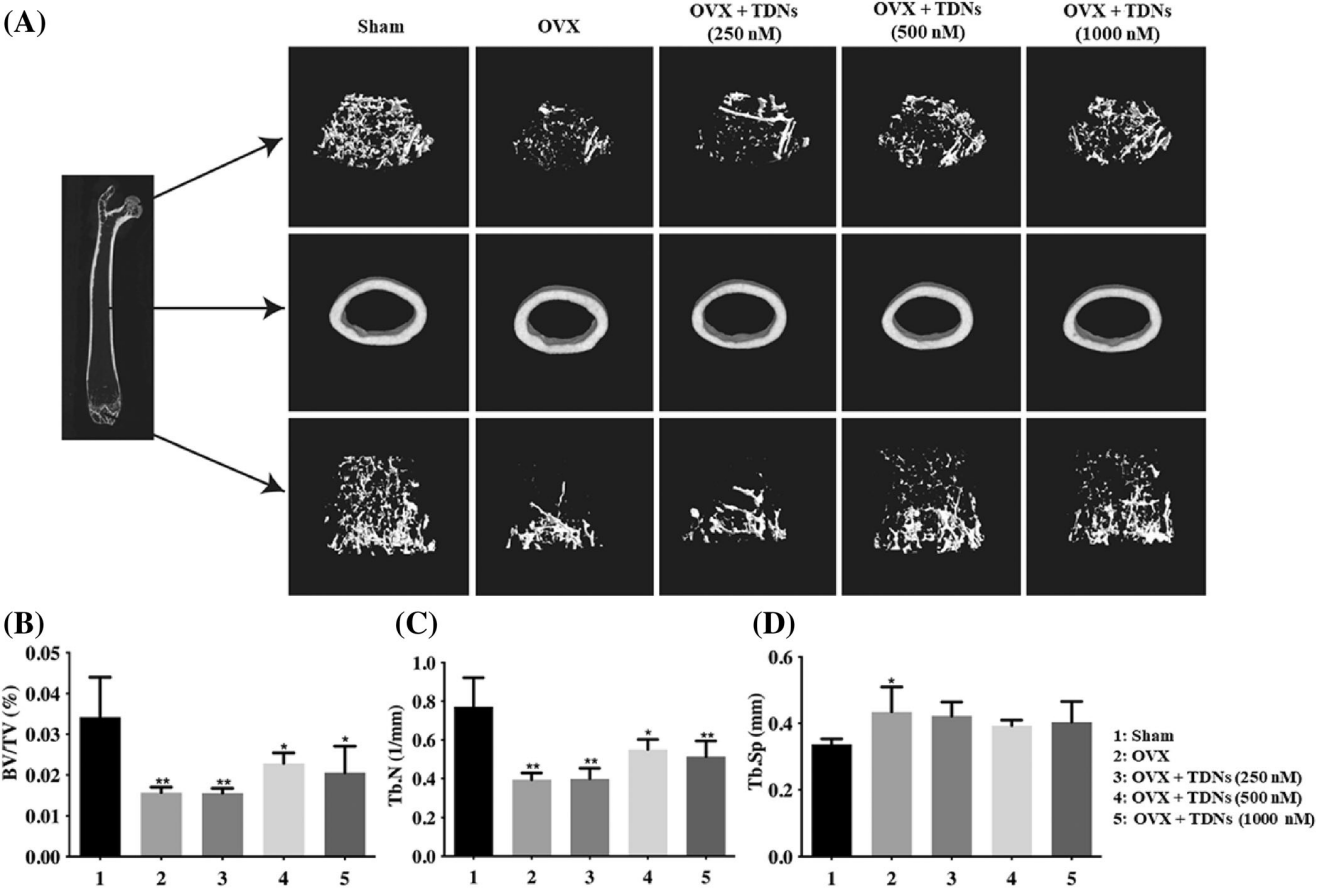
Microcomputed tomography (Micro‐CT) images of the femurs. (A) Micro CT images of the femurs. (B–D) Analysis of bone volume fraction (BV/TV), bone trabecular number (Tb. N), bone separation (Tb. SP) of femurs after treatment with different materials at 2 months, respectively (*p < 0.05, **p < 0.01 vs. Sham).

**FIGURE 3:**
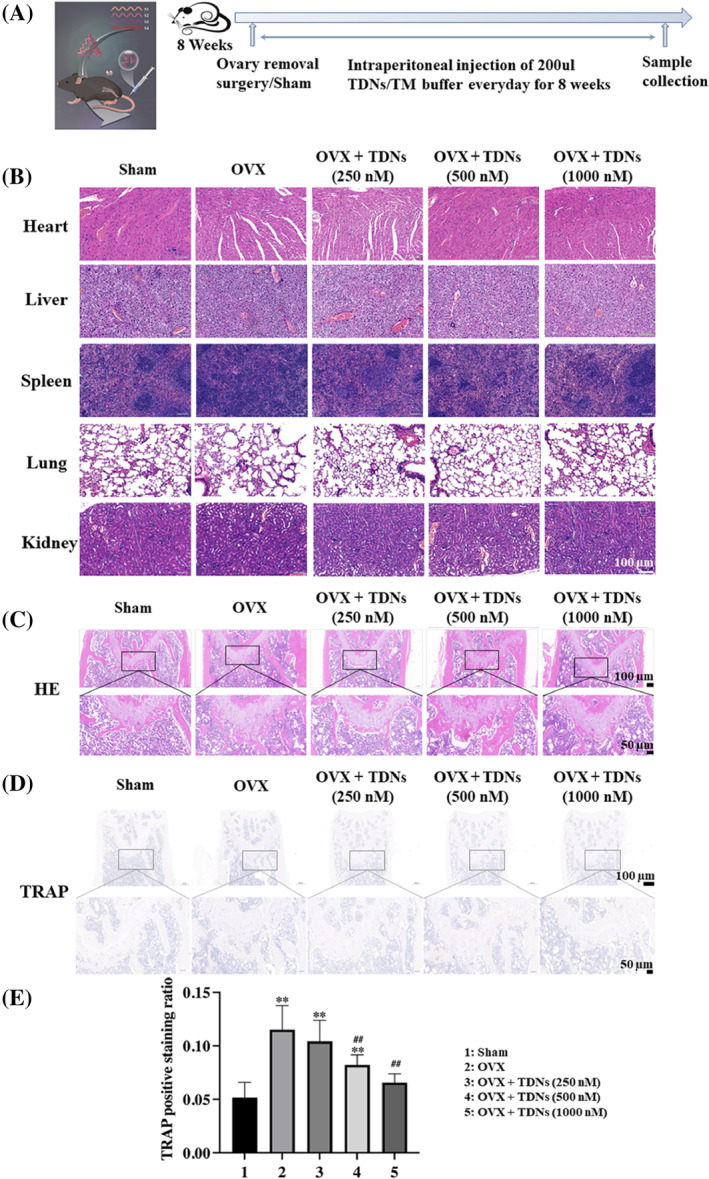
Animal moulding process and staining. (A) Time chart of animal moulding and treatment. (B) Haematoxylin–eosin (HE) staining of heart, liver, spleen, lung, and kidney of each group after 8 weeks of treatment separately (Scale bar: 100 μm). (C) HE staining of the femurs of each group after 8 weeks of treatment (Scale bar: 100 and 50 μm). (D) Tartrate‐resistant acid phosphatase (TRAP) staining of the femurs of each group after 8 weeks of treatment (Scale bar: 100 and 50 μm). (E) Statistical analysis of TRAP staining (*p < 0.05, **p < 0.01 vs. sham; ^#^ *p* < 0.05, ^##^ *p* < 0.01 vs. ovariectomy [OVX]). TDN, tetrahedral DNA nanostructure.

By using the OVX method, an OP mouse model was established, and the use of TDN via tail vein could improve OP (Figure 3A). We found that ovariectomized mice can simulate some physiological changes in OP. Micro‐CT of the femur showed that the bone volume fraction (BV/TV) and bone trabecular number (Tb. N) were significantly decreased, and the bone separation (Tb. SP) was significantly increased (Figure 2A–D). Haematoxylin–eosin (HE) staining of various organs of mice showed that there was no obvious damage to the organs (heart, liver, spleen, lung, kidney) of mice in each group (Figure 3B). HE staining of the femur also showed that the cartilage layer of the joint of the osteoporotic mice was seriously damaged, and there were many bone resorption lacunae in the bone trabecula, as well as many broken and osteoclast lacunae in the bone dense part of the shaft (Figure 3C). As a specific marker enzyme of osteoclasts, TRAP staining significantly increased the expression of TRAP in osteoporotic mice (Figure 3D,E).

### 3.3 TDNs improve OP induced by OVX in mice

After treatment with TDNs, we found that OP in OVX mice was significantly improved. Micro‐CT results showed that after treatment, the bone volume fraction (BV/TV) and bone trabecular number (Tb. N) of mice increased significantly, while the bone separation (Tb. SP) decreased significantly, and the changes were concentration dependent to some extent (Figure 2A–D). At the same time, we also observed changes in bone tissue morphology and structure. The HE staining results of the femur showed that after treatment, both the damage of the cartilage layer in the mice, the osteoclast lacuna in the bone trabecula, and the damage of the bone dense part of the shaft were recovered (Figure 3C). At the same time, TRAP staining, as a specific marker enzyme of osteoclasts, is an important marker for identifying osteoclasts. The results of TRAP staining showed that the content of TRAP‐positive expression in mice decreased after treatment in a concentration‐dependent manner (Figure 3D,E). This suggested that after treatment with TDNs, the osteoclasts of mice were significantly inhibited to avoid further aggravation of OP symptoms.

### 3.4 TDNs regulated the Wnt/β‐Catenin and BMP signalling pathways

**FIGURE 4:**
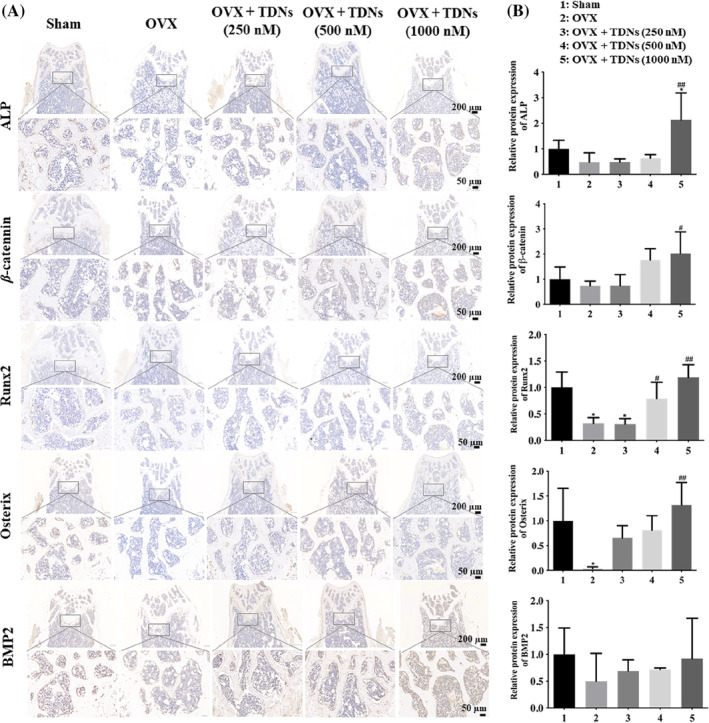
Immunohistochemical staining. (A) Femur haematoxylin–eosin staining of ALP, β‐Catenin, Runx2, Osterix, BMP2, after 8 weeks of treatment separately (Scale bar: 200 and 50 μm). (B) Statistical analysis ALP, β‐Catenin, Runx2, Osterix, BMP2 among five groups separately (*p < 0.05, **p < 0.01 vs. Sham; ^#^ *p* < 0.05, ^##^ *p* < 0.01 vs. ovariectomy [OVX]). TDN, tetrahedral DNA nanostructure.

**FIGURE 5:**
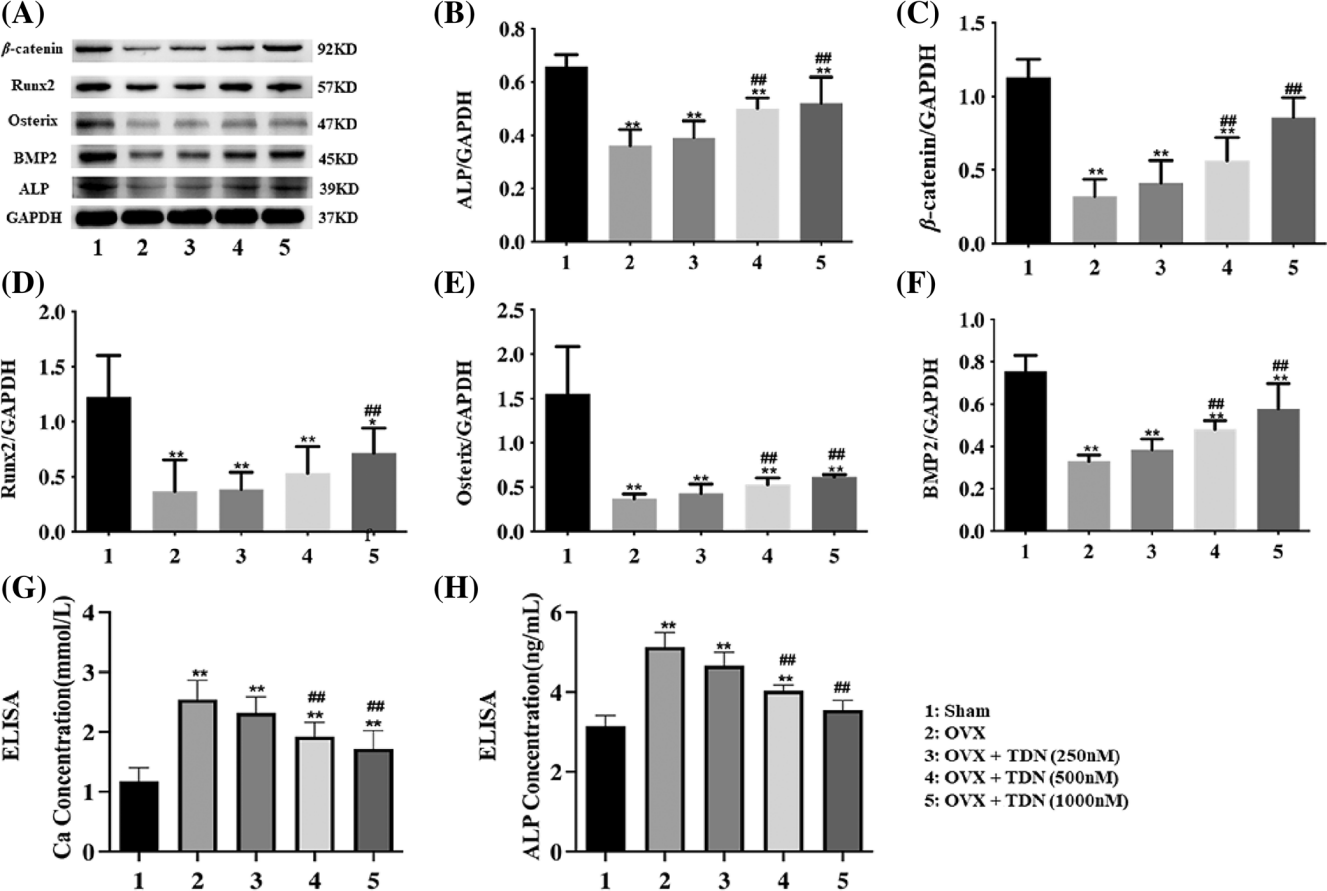
Improve of ovariectomy (OVX)‐induced osteoporosis by tetrahedral DNA nanostructures (TDNs) via Wnt/β⁃Catenin and bone morphogenetic protein (BMP) signalling pathways. (A) Western blotting results of the ALP, β‐Catenin, Runx2, Osterix, BMP2 expression level. (B–F) statistical analysis ALP, β‐Catenin, Runx2, Osterix, BMP2 among five groups separately. (G,H) Enzyme‐linked immunosorbent assay detection of the concentrations of ALP and calcium ions in serum (*p < 0.05, ** *p* < 0.01 vs. sham; ^#^ *p* < 0.05, ^##^ *p* < 0.01 vs. ovariectomy [OVX]).

Immunohistochemical results of femurs suggested that the expression of ALP, β‐Catenin, Runx2, Osterix, and BMP2 decreased significantly in the OP mouse model but significantly increased in a concentration‐dependent manner after treatment with TDNs (Figure 4A,B). WB analysis also showed that these osteogenesis‐related proteins were decreased in OP mice but significantly increased after treatment with TDNs (Figure 5A–F). In addition, the ELISA results of serum showed that the concentrations of ALP and Ca^2+^ in OP mice increased but decreased after treatment (Figure 5G, H). Immunohistochemistry suggested that the positive rate of ALP in bone was high, and ELISA suggested that the free ALP in serum was low. The change in ALP in the femur and serum is inconsistent, which suggests that ALP in serum may be more transferred to bone and participate in bone formation. Therefore, the femur ALP is high, while the serum ALP may be lower, and the bone mineral density of mice is higher. Similarly, the decrease in Ca^2+^ in serum may be related to the greater involvement of calcium salt in the consumption of bone formation. Therefore, the Ca^2+^ in serum may be relatively lower. It can be speculated that after treatment with TDNs, osteogenesis in the bone of mice is promoted to a certain extent, which is helpful to alleviate the symptoms of OP.

The Wnt signalling pathway is a complex protein interaction network, of which the Wnt/β⁃Catenin signalling pathway is a classical Wnt transduction pathway that is closely related to the proliferation and osteogenic differentiation of MSCs and the proliferation, differentiation, and function of osteoblasts and osteoclasts.^19^ Osteoblastic differentiation is the key step of osteogenesis; that is, MSCs undergo a complex process of osteoblastic progenitor cells, osteoblastic precursors, and osteoblasts eventually differentiate into osteoblasts. This process involves many types of intercellular and intracellular signal transduction, such as signal pathways, transcription factors, growth factors, microRNAs, and so forth. Thus, a complete network system of bone metabolism regulation was formed.^20^, ^21^

**FIGURE 6:**
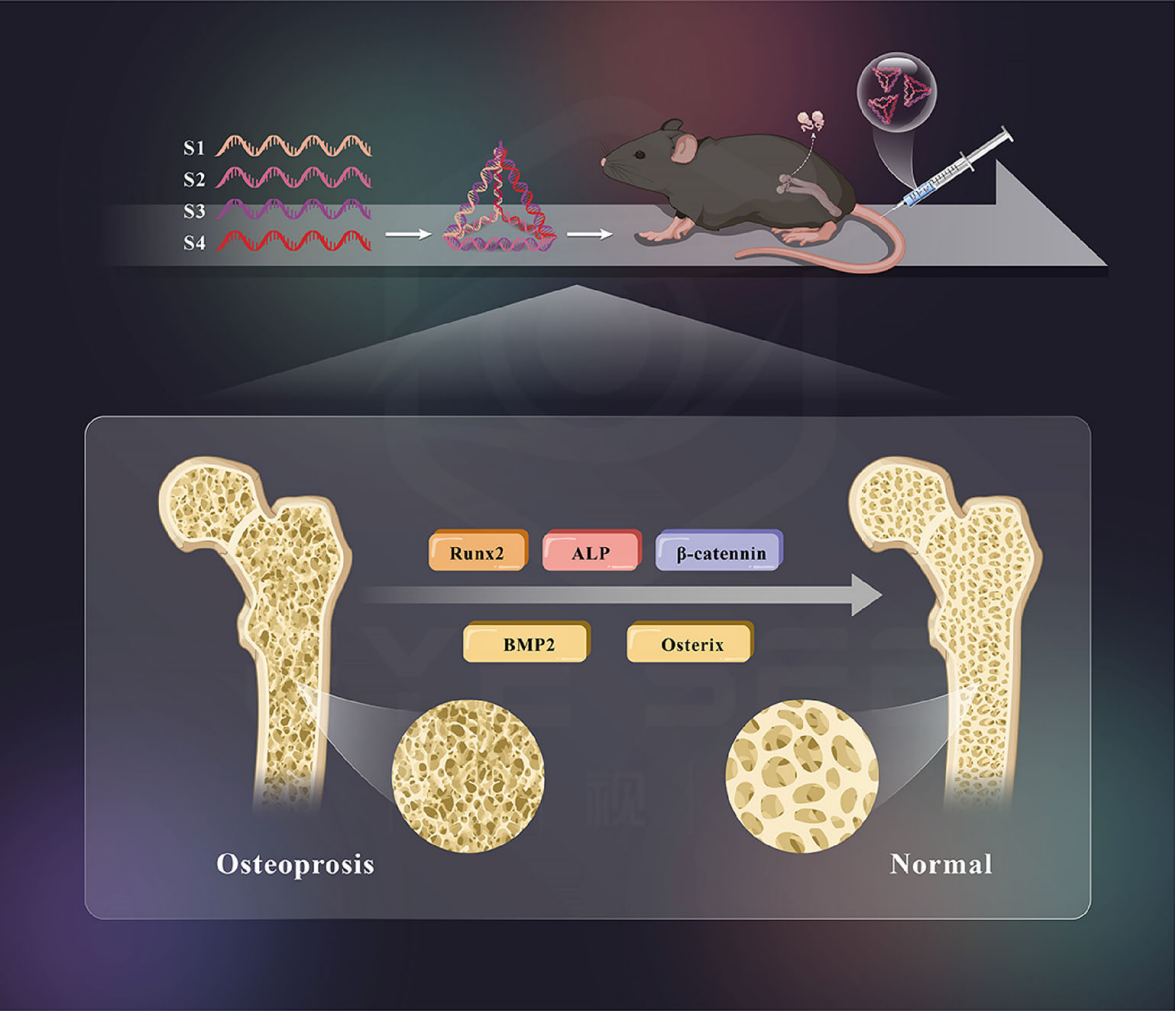
Schematic diagram.

MSCs are a group of cells with differentiation potential that are widely used in immune regulation, tissue repair, organ reconstruction, tissue engineering, drug development, and other research fields.^22^, ^23^ Previous studies have found that their differentiation into osteoblasts is regulated by the Wnt signalling pathway, and inhibition of the Wnt signalling pathway can block the process of osteoblast differentiation and inhibit bone formation. Inducing the expression of Wnt family members may upregulate the expression of osteoblast‐specific genes and promote bone formation.^24^, ^25^, ^26^ Specifically, the osteogenic differentiation of MSCs is regulated by a number of signalling pathways, including the classical Wnt signalling pathway. β‐Catenin activates gene transcription. It can directly or indirectly affect the expression of key osteogenic transcription factors such as Runx2 and Osterix. Therefore, it plays a key role in the process of bone development and regeneration, bone repair, and reconstruction.^27^, ^28^, ^29^ Runx2 is the primary transcription regulator of osteoblast differentiation, and Osterix plays a role downstream. Interestingly, our previous research also found that TDNs can activate the classic Wnt/β‐Catenin signalling pathway, which can increase the activity of ALP and the expression of the osteogenic differentiation‐related protein Runx2 in the process of osteoblastic differentiation. To promote calcium deposition and promote the osteogenic differentiation of adipose MSCs.^17^ In this experiment, we induced an OP animal model and treated the animals with TDNs for 8 weeks (Figure 6). We found that the Wnt/β‐Catenin pathway is activated, the expression level of its specific markers of osteogenic differentiation Runx2 and Osterix is significantly increased, and the level of ALP in bone tissue is also significantly increased.

In addition to the classic Wnt/β‐catenin signalling pathway, bone metabolism is also regulated by a variety of cytokines and growth factors. BMP is one of them. It plays a key regulatory role in bone metabolism. BMP belongs to the transforming growth factor‐β (TGF‐β) superfamily, and TGF‐β members of the superfamily all mediate signal transduction by binding to the two‐receptor system‐type I and type II transmembrane serine/threonine kinase receptors (BMPR‐I, BMPR‐II). Thus, the Smad signalling pathway plays a major role in the process of membrane signal entry into the nucleus.^30^ Loss of the BMP Smad signalling pathway can lead to bone‐related diseases, such as OP.^31^ Additionally, BMP2, as an important subtype of the BMP family, can regulate the expression of Runx2 and Osterix, the key genes of osteoblast differentiation, through the BMP Smad signalling pathway to promote osteoblast differentiation.

In fact, researchers have also found that there is a certain correlation between these signalling pathways. For example, Wnt/β‐Catenin signalling directly induces the expression of BMP2 in osteoblasts through the TCF/LEF response element and enhances the transcriptional activity of BMP2. Additionally, β‐Catenin can enhance the response of MSCs to BMP2, induce their differentiation into osteoblasts, and promote osteogenesis.^32^, ^33^ In general, osteogenesis is a complex physiological process involving multiple systems. Many signalling pathways have been proven to play an important role in this process. This current study found that TDNs may regulate Wnt simultaneously/β⁃Catenin and that the BMP signalling pathway can increase the levels of specific markers of osteogenic differentiation, Runx2, ALP, and Osterix, and play a role in promoting osteogenesis.^34^ This study attempts to conduct a cross‐study from multiple pathways to further reveal the specific mechanism by which TDNs promote osteogenic differentiation and provide evidence for the search for the treatment of OP, a class of osteo‐related diseases.

## 4 CONCLUSION

TDNs activate the Wnt/β‐Catenin and BMP2 signalling pathways and exert therapeutic effects on OP mice. Additionally, prompt Wnt/β‐Catenin and BMP2 signal transduction pathways affect osteoblasts, which may be the target of drugs for treating OP. Drugs developed for these signalling pathways are expected to become promising therapeutic strategies for osteoporosis.

## AUTHOR CONTRIBUTIONS

The article was written through contributions of all authors. All authors have given approval to the final version of the article.

## FUNDING INFORMATION

This study was supported by the National Key R&D Program of China (2019YFA0110600), National Natural Science Foundation of China (82370929 and 81970916), Sichuan Science and Technology Program (2022NSFSC0002), Sichuan Province Youth Science and Technology Innovation Team (2022JDTD0021), Research and Develop Program, West China Hospital of Stomatology Sichuan University (RD03202302), Open Research Program of National Facility for Translational Medicine (Shanghai, TMSK‐2021‐204), and Research Funding from West China School/Hospital of Stomatology Sichuan University (RCDWJS2021‐20).

## CONFLICT OF INTEREST STATEMENT

The authors declare no competing financial interest.

## Data Availability

The data that support the findings of this study are available from the corresponding author upon reasonable request.
